# Structural Requirements of Benzofuran Derivatives Dehydro-*δ*- and Dehydro-*ε*-Viniferin for Antimicrobial Activity Against the Foodborne Pathogen *Listeria monocytogenes*

**DOI:** 10.3390/ijms21062168

**Published:** 2020-03-21

**Authors:** Giorgia Catinella, Luce M. Mattio, Loana Musso, Stefania Arioli, Diego Mora, Giovanni Luca Beretta, Nadia Zaffaroni, Andrea Pinto, Sabrina Dallavalle

**Affiliations:** 1 Department of Food, Environmental and Nutritional Sciences (DeFENS), University of Milan, Via Celoria 2, 20133 Milan, Italy; giorgia.catinella@unimi.it (G.C.); luce.mattio@unimi.it (L.M.M.); loana.musso@unimi.it (L.M.); stefania.arioli@unimi.it (S.A.); diego.mora@unimi.it (D.M.); sabrina.dallavalle@unimi.it (S.D.); 2 Molecular Pharmacology Unit, Department of Applied Research and Technological Development, Fondazione IRCCS Istituto Nazionale Tumori, Via Amadeo 42, 20133 Milan, Italy; giovanni.beretta@istitutotumori.mi.it (G.L.B.); nadia.zaffaroni@istitutotumori.mi.it (N.Z.)

**Keywords:** viniferin derivatives, benzofuran nucleus, antimicrobials, *Listeria monocytogenes*

## Abstract

In a recent study, we investigated the antimicrobial activity of a collection of resveratrol-derived monomers and dimers against a series of foodborne pathogens. Out of the tested molecules, dehydro-*δ*-viniferin and dehydro-*ε*-viniferin emerged as the most promising derivatives. To define the structural elements essential to the antimicrobial activity against the foodborne pathogen *L. monocytogenes* Scott A as a model Gram-positive microorganism, the synthesis of a series of simplified benzofuran-containing derivatives was carried out. The systematic removal of the aromatic moieties of the parent molecules allowed a deeper insight into the most relevant structural features affecting the activity. While the overall structure of compound **1** could not be altered without a substantial loss of antimicrobial activity, the structural simplification of compound **2** (minimal inhibitory concentration (MIC) 16 µg/mL, minimal bactericidal concentration (MBC) >512 µg/mL) led to the analogue **7** with increased activity (MIC 8 µg/mL, MBC 64 µg/mL).

## 1. Introduction

Benzofuran derivatives are an important class of heterocyclic compounds, occupying a place in numerous bioactive natural products. Over time, they have attracted the attention of many researchers due to the broad scope of their activity, which includes anticancer, antimicrobial, immunomodulatory, antioxidant and anti-inflammatory properties [1,2].

In recent years, the benzofuran scaffold has emerged as a pharmacophore of choice for designing antimicrobial agents, and numerous achievements have revealed that benzofuran-based compounds have an extensive potential in this field [3].

**Figure 1:**
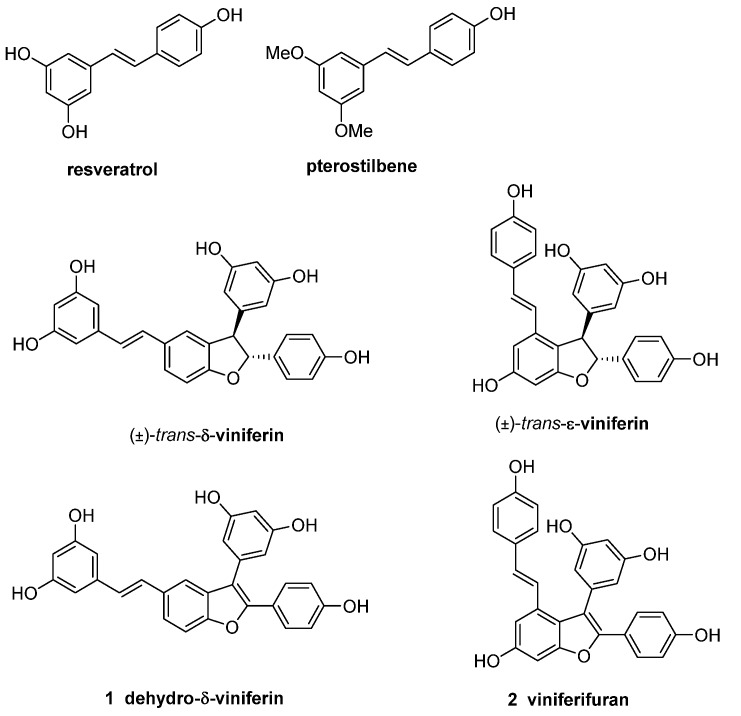
Structures of resveratrol and representative resveratrol-derived monomers and dimers.

As a part of our efforts in the search for new antimicrobial agents, we have recently evaluated the antimicrobial activity of a collection of resveratrol-derived monomers (e.g., resveratrol, pterostilbene) and dimers (e.g., *trans*-*δ*-viniferin, *trans*-*ε*-viniferin, dehydro-*δ*-viniferin **1**, viniferifuran **2**), which were screened as single molecules against a panel of foodborne pathogens (Figure 1) [4].

The most promising dimeric compound, dehydro-*δ*-viniferin **1**, was endowed with significant antibacterial activity against Gram-positive bacteria. It was verified that the activity of **1** against *L. monocytogenes* was achieved by damaging the cytoplasmic membrane with significant membrane depolarization, a loss of membrane integrity, and severe morphological changes. The dimeric compound **2** showed significant antimicrobial activity as well [4].

**Figure 2:**
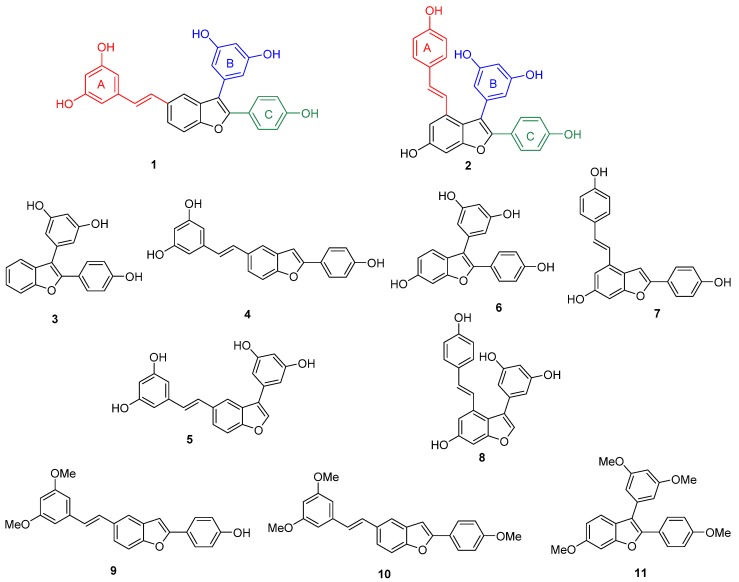
Structures of compounds **1** and **2** and of the simplified analogues **3**–**8** and **9**–**11**.

These promising results prompted us to explore **1** and **2** as platforms in the search for new antimicrobial scaffolds. To provide a deeper insight into the activity of these compounds, we planned to perform preliminary structure–activity relationship (SAR) studies. The unique structural features of compounds **1** and **2**, both containing a versatile benzofuran core structure, make them amenable for the generation of differently substituted analogues. To shed light on the minimal structural features which retain antimicrobial activity, we designed a small collection of simplified derivatives of compounds **1** and **2** by a systematic removal of the moieties linked to the benzofuran rings (groups A, B, C) (Figure 2), and we tested all the derivatives against *Listeria monocytogenes* as a representative foodborne Gram-positive bacterium, which was found to be sensitive to compounds **1** and **2** [4].

Therefore, we designed the three possible simplified analogues of compound **1** (compounds **3**–**5**) and the three possible simplified analogues of compound **2** (compounds **6**–**8**) (Figure 2). In addition, we designed three representative analogues (**9**–**11**) containing the -OMe groups in place of the phenolic -OH on the aromatic rings.

The evaluation of the antimicrobial activity of the compounds provided an integrated overview of the structural determinants for the activity against the foodborne pathogenic species *L. monocytogenes* Scott A.

## 2. Results

In the present SAR study, different synthetic approaches were set up depending on the nature of the benzofuran substitution pattern.

**Scheme 1:**
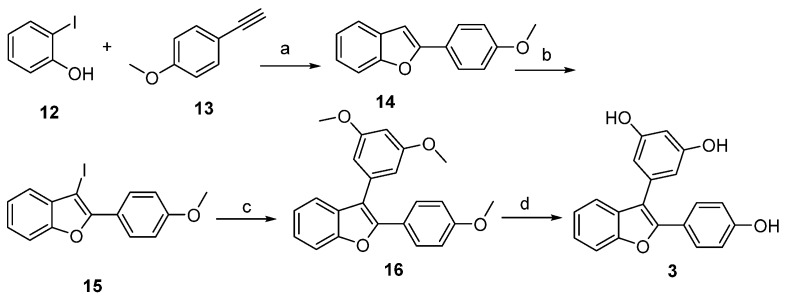
Reagents and conditions: (a) (i) PdCl_2_(PPh_3_)_2_ (5% mol), CuI (3% mol), TEA:THF 1:1, N_2_, 40 °C, 40 min; (ii) ACN, 100 °C, 90 min, 50%; (b) NIS, p-TsOH, ACN, N_2_, overnight, 74%; (c) (3,5-dimethoxyphenyl)boronic acid, K_2_CO_3_, PdCl_2_(dppf)·CH_2_Cl_2_, THF:H_2_O 1:1, MW, 70 °C, 30 min, 83%; d) BBr_3_, DCM, 0 °C to rt, overnight, 61%.

Initially, we focused on the synthesis of the simplified analogues of compound **1**. Compound **14** was obtained by a Cu-catalyzed tandem Sonogashira coupling–cyclization reaction starting with 2-iodophenol **12** and 1-ethynyl-4-methoxybenzene **13** [5,6]. The iodination of **14** with NIS gave compound **15** in a 74% yield [7]. Suzuki coupling of **15** with (3,5-dimethoxyphenyl)boronic acid gave the permethylated intermediate **16**, which, after the deprotection of the phenolic -OH with BBr_3_, afforded 2,3-disubstituted benzofuran **3** (Scheme 1).

**Scheme 2:**
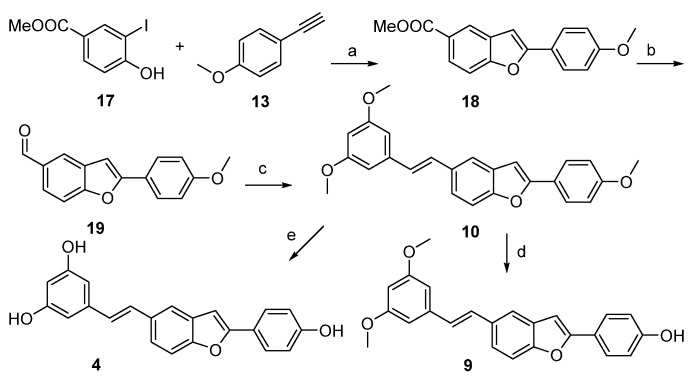
Reagents and conditions: (a) (i) PdCl_2_(PPh_3_)_2_ (5% mol), CuI (3% mol), TEA:THF 1:1, N_2_, 40 °C, 40 min; (ii) ACN, 100 °C, 90 min, 62%; (b) (i) LiAlH_4_, THF, N_2_, 0 °C, 10 min; (ii) DMP, DCM, 0 °C 15 min, rt 90 min, 85%; (c) diethyl (3,5-dimethoxyphenyl)phosphonate, NaH, THF, MW, 120 °C, 30 min, 76%; (d) BBr_3_, DCM, 0 °C to rt, overnight, 60%; (e) BCl_3_, TBAI, DCM, N_2_, 0 °C to rt, 6 h, 35%.

The synthesis of compound **4** was based on the same key Cu-catalyzed tandem Sonogashira coupling-cyclization described above for the construction of the 2-aryl-substituted benzofuran ring (Scheme 2). However, in this case the iodophenol should have a suitable moiety in position 4 to install the styryl functionality. Thus, compound **17** was reacted with **13** to obtain the ester **18**, which was then converted into the corresponding aldehyde **19** by an LiAlH_4_ reduction, followed by a Dess–Martin periodinane oxidation. A Wittig–Horner olefination with diethyl (3,5-dimethoxyphenyl)phosphonate under microwave irradiation afforded **10**. The final deprotection step of all the phenol groups was quite troublesome. Previous works reported that the demethylation of the stilbenoid derivatives was achieved using BBr_3_ in CH_2_Cl_2_ [8]. However, when applying these conditions to compound **10**, only the partially demethylated compound **9** was obtained. We, therefore, decided to explore the alternative route reported by Vo et al. [9], based on the use of boron trichloride/tetra-*n*-butylammonium iodide (BCl_3_/TBAI). Actually, the reagent was found to be more effective than boron tribromide and allowed us to obtain the demethylated compound **4**. A semi-preparative HPLC purification was required to obtain the pure compound in a 35% yield.

**Scheme 3:**
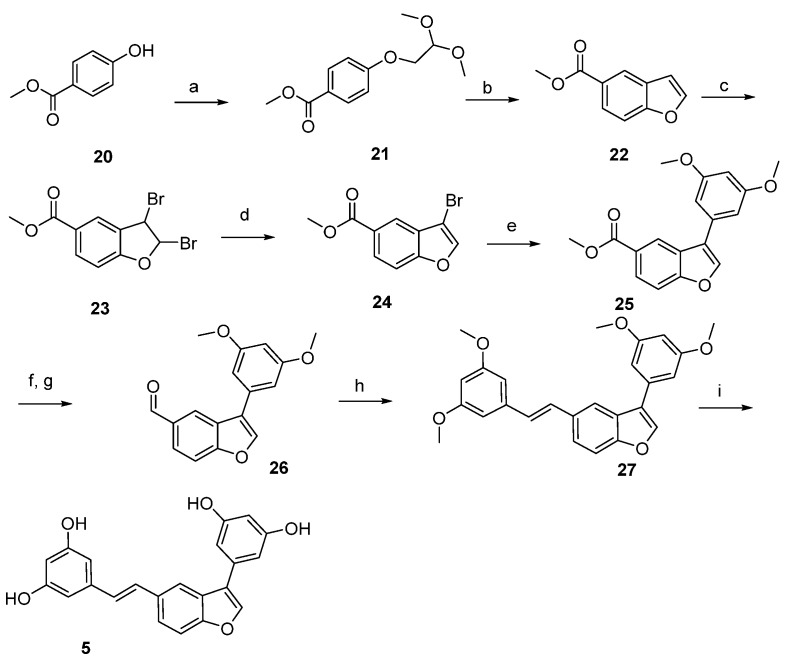
Reagents and conditions: (a) 2-bromo-1,1-dimethoxyethane, Cs_2_CO_3_, MeCN, reflux, 3 days, 61%; (b) Amberlyst-15, toluene, reflux, 6 h, 51%; (c) Br_2_, DCM, 0 °C to rt, 75 min, 82%; (d) KOH, MeOH, THF, 0 °C, 20 min, 82%; (e) (3,5-dimethoxyphenyl)boronic acid, Pd(PPh_3_)_4_, Na_2_CO_3_, DME:H_2_O 5:1, 80 °C, overnight, 74%; (f) LiAlH_4_, THF, 0 °C, 10 min, 97%; (g) DMP, DCM, 0 °C to rt, 90 min, 78%; (h) diethyl (3,5-dimethoxyphenyl)phosphonate, NaH, THF, MW, 120 °C, 30 min, 80%; (i) BBr_3_, DCM, 0 °C to rt, overnight, 14%.

The functionalization of positions 3 and 5 of the benzofuran ring required a different synthetic approach (Scheme 3). The heterocyclic core was synthesized efficiently from **20** by the sequence of alkylation with bromoacetaldehyde dimethylacetal and cyclodehydration using Amberlyst-15, following a procedure described by Liu et al. [10]. The introduction of a bromine in position 3 was obtained by the bromination of **22** followed by a treatment with KOH in MeOH [11]. The Suzuki coupling of compound **24** with (3,5 dimethoxyphenyl) boronic acid afforded compound **25**. LiAlH_4_ reduction followed by a Dess–Martin periodinane oxidation gave the aldehyde **26**, which underwent a Wittig–Horner olefination with diethyl(3,5-dimethoxyphenyl)phosphonate under microwave irradiation at 120 °C to give compound **27**. Again, the critical step was the final deprotection of the phenolic groups. After several attempts, compound **5** was obtained in a 14% yield by a treatment with BBr_3_ and a troublesome purification by flash chromatography.

Successively, we focused on the analogues of compound **2**. Kim and Choi [12] employed a versatile and mild procedure to construct a 3-arylbenzofuran by the cyclization of the corresponding *β*-aryloxyketone using Bi(OTf)_3_. Following this protocol, compound **30** was prepared by the reaction of *m*-methoxyphenol with the α-bromoketone **29**, on its turn obtained by treatment of 3,5-dimethoxyacetophenone **28** with CuBr_2_. The *β*-aryloxyketone **30** was subjected to a cyclodehydration reaction with Bi(OTf)_3_, involving an intramolecular Friedel–Craft acylation followed by dehydration to give the desired benzofuran **31**. The same synthetic protocol was applied to the ketone **32** to obtain the benzofuran **33**. In both cases, the intramolecular cyclization took advantage of the presence of the electron-donating OMe group.

**Scheme 4:**
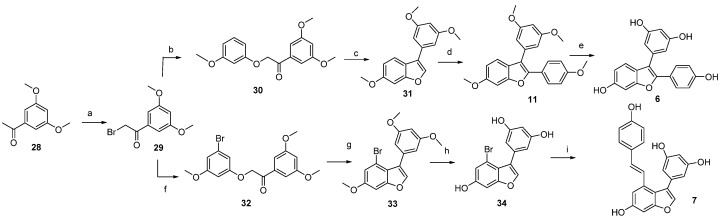
Reagents and conditions: (a) CuBr_2_, EtOAc:CHCl_3_, reflux, overnight, 67%; (b) m-methoxyphenol, K_2_CO_3_, acetone, N_2_, reflux, 2 h, 90%; (c) Bi(OTf)_3_, DCM, N_2_, reflux, overnight, 43%; (d) p-methoxybromobenzene, Pd(OAc)_2_, PCy_3_HBF_4_, K_2_CO_3_, pivalic acid, DMA, N_2_, 100 °C, 20 h, 80%; (e) BBr_3_, DCM, 0 °C to rt, overnight, 52%; (f) 3-bromo-5-methoxyphenol, K_2_CO_3_, acetone, N_2_, reflux, 2 h, 89%; (g) Bi(OTf)_3_, DCM, N_2_, reflux, overnight, 83%; (h) BBr_3_, DCM, 0 °C to rt, overnight, 91%; (i) p-hydroxystyrene, Pd(OAc)_2_, TEA, dppp, DMF, N_2_, 120 °C, 20 h, 80%.

The installation of a further aromatic ring on the C-2 of compound **31** was obtained by direct arylation by using Pd(OAc)_2_ and P(Cy)_3_·HBF_4_ [13]. The deprotection of the derivative **11** with BBr_3_ afforded compound **6** in a 52% yield (Scheme 4).

Conversely, to obtain compound **7**, it was necessary to introduce the styryl moiety in position 4. To avoid the formation of side products due to the reactivity of the styryl double-bond with the deprotecting reagent, we first treated compound **33** with BBr_3_ to afford compound **34** in a 91% yield. Finally, a Heck coupling with *p*OH styrene gave the desired scaffold **7**.

**Scheme 5:**
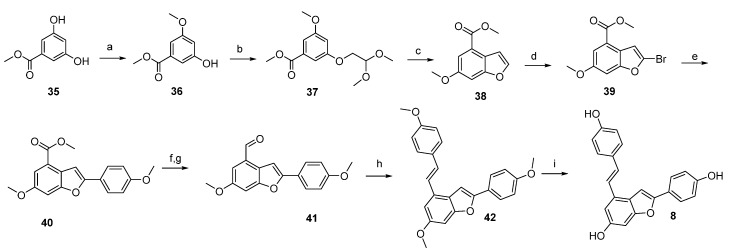
Reagents and conditions: (a) K_2_CO_3_, CH_3_I, DMF, rt, 45h, 35%; (b) 2-bromo-1,1-dimethoxyethane, Cs_2_CO_3_, CH_3_CN, reflux, 72 h, 67%; (c) Amberlyst-15, C_6_H_5_Cl, 120 °C, 3 h, 63%; (d) NBS, DMF cat., ClCH_2_CH_2_Cl, 75 °C, 3 h, 80%; (e) (4-methoxyphenyl)boronic acid, Pd(PPh_3_)_4_, K_2_CO_3_, DMF, 70 °C, overnight, 91%; (f) LiAlH_4_, DCM, 0 °C, 10 min, 89%; g) DMP, DCM, 0 °C to rt, 90 min, 97%; (h) diethyl (4-methoxybenzyl)phosphonate, NaH, dry THF, 120 °C - MW, 30 min, 54%; (i) BBr_3_, 0 °C to rt, 6 h, 24%.

The last simplified analogue was obtained as reported in Scheme 5. Compound **35** was methylated to give compound **36**, following the procedure described by Iino [14]. However, in our study, the selective methylation reaction also gave 40% of a dialkoxy compound, which needed to be separated by column chromatography. The benzofuran derivative **38** was then obtained by the sequence of alkylation with bromoacetaldehyde dimethylacetal and cyclodehydration using Amberlyst-15, as described before. A treatment with NBS afforded 2-bromobenzofuran **39**, which was isolated in a high yield as the only isomer when we used dichloroethane as the solvent and DMF as the catalyst, as described by Liu [10]. A Suzuki coupling followed by the conversion of the ester group into aldehydes, as previously reported, led to compound **41**, which was then subjected to an olefination reaction to give compound **42**. Finally, the deprotection of the -OMe groups gave the simplified compound **8**.

The synthesized molecules **1**–**11** were tested against the foodborne pathogen *L. monocytogenes* Scott A, after being recognized as one of the most sensitive species as a result of our previous screening [4]. The results are reported in Table 1. The minimal inhibitory concentration (MIC) and minimal bactericidal concentration (MBC) values confirmed the higher sensitivity of *L. monocytogenes* to compound **1** (MIC 2 μg/mL, MBC 16 μg/mL) rather than to compound **2** (MIC 16 μg/mL, MBC >512 μg/mL). In general, the structural modifications of compounds **1** and **2** negatively interfered with their antimicrobial activity (Table 1). However, compound **7**, a simplified analogue of compound **2**, showed an evident decrease in its MIC (8 μg/mL) and MBC (64 μg/mL) values, reflecting an improved antimicrobial activity.

## 3. Discussion

The rapid development of the resistance of bacterial pathogens against antimicrobial agents still represents an important problem [16,17]. To overcome this drawback, the identification and development of novel scaffolds is imperative.

Stilbenoids are an intriguing structural class of natural polyphenols characterized by a diverse array of biological properties [18]. Among stilbenoids, resveratrol and the dimethyl derivative pterostilbene have shown promising antimicrobial activity [19,20].

Recently, we investigated a focused collection of resveratrol-derived compounds, including resveratrol-derived monomers and dimers, which were screened against a series of foodborne pathogens [4]. The collected evidence indicated that most of the compounds were endowed with a significant antimicrobial activity against Gram-positive bacteria, with **1** and **2** being the most potent molecules of the series.

To shed light on the SAR of these compounds, and to understand whether structural modifications could be exploited in the development of more potent congeners, we synthesized the simplified derivatives of **1** and **2**. Importantly, the selective removal of the aromatic rings from compound **1′**s skeleton significantly reduced antimicrobial activity. In fact, compared to the parent compound **1**, the derivatives **3**, **4** and **5**, obtained from the removal of the moieties in positions 5, 3 and 2, respectively, had significantly lower activity. In particular, the removal of the ring B (derivative **4**), resulted in a completely inactive compound (MIC 256 μg/mL, MBC >512 μg/mL). This finding suggests that the three phenolic portions A, B, and C present in the parent compound **1** are all important and synergic for antimicrobial activity.

Conversely, SAR studies on the analogues of compound **2** showed that the removal of the ring B increased the activity of the derivative. Indeed, compound **7** was more potent than **2** (MIC 8 μg/mL, MBC 64 μg/mL vs. MIC 16 μg/mL, MBC >512 μg/mL), whereas compounds **6** and **8**, obtained by the selective removal of the moieties C and A, respectively, showed four-fold lower activity, only in terms of MIC, compared to the parent compound **2** (MIC 64 μg/mL vs. 16 μg/mL). It is interesting to notice that the most active compound of this series, compound **7**, has three phenolic portions with a spatial orientation similar to that of compound **1**. Interestingly, the introduction of a hydroxy group on the benzofuran skeleton of compound **3** (**6** vs. **3**), caused a four-fold reduction in activity (MIC 64 μg/mL, MBC >512 μg/mL vs. MIC 16 μg/mL, MBC 64 μg/mL). The replacement of the phenolic hydroxyl groups with the methoxy groups was, in any case, deleterious, giving inactive compounds (compounds **9**, **10**, **11**). Notably, not only the spatial orientation but also the distance between the substituents of the rings seemed to play a role. In fact, compound **9** differed from pterostilbene only in a longer spacer connecting the two substituted rings (the benzofuran core). This difference results in a drop in compound activity (MIC and MBC >512 μg/mL vs. MIC 64 μg/mL, MBC 128 μg/mL).

Overall, the results confirm that the shape and geometry of the molecules play a key role in the antimicrobial activity. Indeed, it is well documented that the hydroxy groups in polyphenolic compounds can interact with the bacterial cell membrane [21,22], and that the relative position of the -OH groups on the phenolic nucleus strongly influences the antibacterial efficacy [23,24].

To evaluate the potential toxicity of the compounds on healthy human cells, the antiproliferative activity of the synthesized molecules, together with the natural compounds resveratrol and pterostilbene, was evaluated on normal skin fibroblast WS1 cells (Table 1). The concentration of compound causing 50% cell growth inhibition (IC_50_) of resveratrol was > 200 μM, whereas the IC_50_ of all the other compounds ranged from 33 to >100 μM. A comparison with the MIC of the compounds against *L. monocytogenes* Scott A (μM concentration, Table 1) showed that **1** has a very good profile of selectivity. Indeed, cytotoxic effects were observed at a concentration ~10-fold higher than the lowest concentration of the compound that prevents the visible growth of bacteria. Moreover, the simplified analogues **3**, **5**, **7** displayed cytotoxic activity in a range of concentration two-fold higher than the MIC values.

Overall, the collected data identified the scaffolds of compounds **1** and **7** as the most promising for further developments.

## 4. Materials and Methods

### 4.1. Chemical Synthesis

The procedures for the synthesis and characterization data for the various derivatives and intermediates are detailed in the Supplementary Materials.

### 4.2. Evaluation of the Minimal Inhibitory Concentration (MIC) and Minimal Bactericidal Concentration (MBC)

All synthesized derivatives were stored in dry form at −20 °C and dissolved in DMSO at a final concentration of 4.096 mg/mL prior to their use in the MIC and MBC determination. Briefly, *L. monocytogenes* was cultivated in Brain Hearth Infusion (BHI) broth (Sigma, Italy) at 37 °C for 24 h under a shaking condition (200 rpm). After the growth, the cells were diluted in BHI to 0.2 OD 600 nm and then used for the 96-well plate inoculum. The concentrations tested ranged from 1 up to 512 µg/mL. The assay was carried out in triplicate in a 96-well plate, according to Mattio et al., 2019 [4].

### 4.3. Cytotoxicity on Human Skin Normal WS1 Fibroblast Cells

The normal human skin WS1 fibroblast cells (ATCC CRL-1502) were cultured in Eagle’s Minimum Essential Medium plus 10% fetal bovine serum at 37 °C and 5% CO_2_.

Cytotoxic potency was assessed by a growth inhibition assay (CellTiter 96^®^ AQueous One Solution Cell Proliferation Assay MTS, Promega). The cells were seeded in a 96-well plate, and 24 h later were exposed to the compounds (concentration range 1−200 μM). After 48 h of exposure, 20 μL of 3-(4,5-dimethylthiazol-2-yl)-5-(3-carboxymethoxyphenyl)-2-(4-sulfophenyl)-2H-tetrazolium salt was added to each well. The absorbance was measured using a FLUOstar OPTIMA plate reader (BMG Labtech GmbH, Offenburg, Germany) at 492 nm after 4 h of incubation at 37 °C in 5% CO_2_. The IC_50_ was defined as the drug concentration causing 50% cell growth inhibition, determined by the dose−response curves. Experiments were performed in triplicate.

## 5. Conclusions

In the present study, we gained further insight into the properties of the viniferin analogues and reported the structural requirements which are fundamental in the antimicrobial activity of the derivatives **1** and **2**. Moreover, we showed that, although less active than the parent compound **1**, the simplified derivatives **3** and **5** retained antimicrobial activity. In addition, we were pleased to find that the simplification strategy adopted for compound **2** was successful. Indeed, compound **7**, which showed two-fold higher potency compared with **2**, represents an intriguing new versatile scaffold for further derivatization.

The cytotoxic profile observed on normal human cells was interesting. Indeed, on the WS1 fibroblasts, compounds **1**, **3**, **5**, **7** showed cytotoxic effects at concentrations at least two-times higher than that observed on *Listeria monocytogenes*. Among these, compound **1** resulted as the most selective of the series.

In conclusion, this study identified dehydro-*δ*-viniferin **1** and compound **7** (the simplified derivative of viniferifuran **2**) as promising scaffolds for the development of nature-inspired antimicrobials active against foodborne pathogenic species.

## Figures and Tables

**Table 1:** Antimicrobial activity of synthesized compounds against *L. monocytogenes* Scott A ^a^ and cytotoxic activity on WS1 ^b^.

Compound	*L. monocytogenes* Scott A	*L. monocytogenes* Scott A	WS1
	MIC (MBC) µg/mL ^a^	MIC (µM) ^a^	IC_50_ (µM) ^b^
resveratrol	200 (-) ^c^	876	>200
pterostilbene	64 (128)	250	57 ± 10
**1** dehydro-*δ*-viniferin	2 (16)	4.42	37.0 ± 1.4
**2** viniferifuran	16 (>512)	35.4	33.0 ± 1.4
**3**	16 (64)	50.3	98.7 ± 1.8
**4**	256 (>512)	743	97.8 ± 3.0
**5**	16 (64)	44.4	96.8 ± 4.5
**6**	64 (>512)	191	98.5 ± 2.0
**7**	8 (64)	23.2	45.0 ± 1.2
**8**	64 (>512)	178	85.0 ± 4.6
**9**	>512 (>512)	1370	95.0 ± 2.3
**10**	>256 (>256)	662	>100
**11**	>256 (>256)	656	>100
Chlorhexidine	8 (32)	15.8	-

^a^ MIC is the minimal inhibitory concentration inhibiting the growth; MBC (in brackets) is the minimal bactericidal concentration. ^b^ IC_50_ is defined as the concentration of compound causing 50% cell growth inhibition. Twenty-four hours after seeding, cells were exposed for 48 h to the compounds and cytotoxicity was measured using MTS assay. Data represent mean values ± SD of three independent experiments. ^c^ *L. monocytogenes* LMG 16,779 [15].

## Supplementary Materials

Supplementary Materials can be found at https://www.mdpi.com/1422-0067/21/6/2168/s1.
