# Supplementary material for: Structural Requirements of Benzofuran Derivatives Dehydro-δ- and Dehydro-ε-Viniferin for Antimicrobial Activity Against the Foodborne Pathogen Listeria monocytogenes
Source: Int J Mol Sci. 2020 Mar 21;21(6):2168. doi: 10.3390/ijms21062168 (PMC7139904; doi:10.3390/ijms21062168)
Supplement: Supplementary file 1 [file ijms-21-02168-s001.pdf]

Supporting Information

Structural requirements of benzofuran derivatives dehydro-*δ*- and dehydro-*ε*-viniferin for antimicrobial activity against the foodborne pathogen *Listeria monocytogenes*

Giorgia Catinella, Luce Micaela Mattio, Loana Musso, Stefania Arioli, Diego Mora, Giovanni Luca Beretta, Nadia Zaffaroni, Andrea Pinto* and Sabrina Dallavalle.

* Correspondence: andrea.pinto@unimi.it

Table of Content:

General information. 1
Synthesis of compound **3**. 3
Synthesis of compound **4**. 5
Synthesis of compound **5**. 8
Synthesis of compounds **6** and **7** 12
Synthesis of compound **8**. 16
References 20

Abbreviations

EtOAc= Ethyl acetate; CHX= Cyclohexane; DCM= Dichloromethane; DMA= Dimethylacetamide; $Bi(OTf)_3$= Bismuth(III) triflate; $PCy_3{\cdot}HBF_4$= Tricycohexylphosphine tetrafluoroborate; FC= Flash Chromatography; $Pd(OAc)_2$= Palladium(II) acetate; dppp= 1,3-bis(diphenylphosphino); TEA= Triethylamine; DMF= Dimethylformamide; ACN= Acetonitrile; NBS= N-bromosuccinimide; MeOH= Methanol; iPrOH: Isopropanol; DME= Dimethoxyethane; THF=Tetrahydrofuran.

General information.

All reagents and solvents were reagent grade or were purified by standard methods before use. Unless otherwise specified, chemicals were from Sigma-Aldrich (Milan, Italy). All reagents and solvents were reagent grade or were purified by standard methods before use. Melting points were determined on a model B-540 Büchi apparatus and are uncorrected. Preparative HPLC was performed with a Kromasil 5-AmyCoat column (21.2 x 250 mm), fitted to a 1525 Extended Flow Binary HPLC pump and a Waters 2489 UV/Vis detector (both from Waters, Milan, Italy). NMR data were acquired using a Varian Mercury-300 MHz spectrometer (Varian, Palo Alto, CA, USA) and a Bruker Avance AV600 spectrometer-600 MHz (Bruker, Rheinstetten, Germany). Chemical shifts (δ values) and coupling constants (*J* values) are given in ppm and Hz, respectively. All reactions requiring anhydrous conditions were performed under a positive nitrogen flow and all glassware were oven dried and/or flame dried. Isolation and purification of the compounds were performed by flash column chromatography on silica gel 60 (230-400 mesh). Analytical thin-layer chromatography (TLC) was conducted on TLC plates (silica gel 60 F254, aluminium foil). Compounds on TLC plates were detected under UV light at 254 and 365 nm. Procedures for synthesis, isolation, and characterization data for the various simplified products are detailed in the following Experimental Procedures section, together with literature references.

Synthesis of compound 3.

- **2-(4-methoxyphenyl)benzofuran (14).**

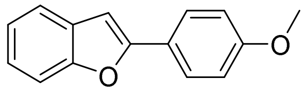

A solution of 2-iodophenol **12** (0.45 mmol, 1 eq.), 1-ethynyl-4-methoxybenzene **13** (0.54 mmol, 1.2 eq.), CuI (0.013 mmol, 0.03 eq.) and $PdCl_2(PPh_3)_2$ (0.02 mmol, 0.05 eq.) in TEA (9.09 mmol, 20 eq.) and THF (1.2 mL) was degassed and warmed to reflux (40 °C) under N_2 for 40 min. After addition of ACN (2.4 mL) the reaction mixture was warmed to 100 °C for 90 min., then it was allowed to cool at room temperature and concentred under reduced pressure. The residue was purified by flash chromatography (FC) with CHX:EtOAc (98:2) as eluent to give the desired product. Yield: 50%, white solid, m.p.: 148-150 °C. ^{1}H NMR: (300 MHz, $CDCl_3$) δ 7.85-7.78 (m, 2H), 7.59-7.49 (m, 2H); 7.31-7.19 (m, 2H), 7.02- 6.96 (m, 2H), 6.90 (d, *J* = 0.9 Hz, 1H), 3.87 (s, 3H). ^{13}C NMR in accordance with literature report. [1]

- **3-Iodo-2-(4-methoxyphenyl)benzofuran (15).**

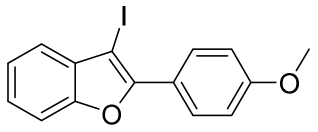

To the suspension of **14** (0.22 mmol, 1 eq.) in CH_3CN (6.7 mL) NIS (0.22 mmol, 1 eq.) and p-toluensulfonic acid (0.22 mmol, 1 eq.) were added. The mixture reaction was stirred overnight under nitrogen at room temperature. Then it was diluted with EtOAc, washed with a saturated solution of $NaHCO_3$, with a 10% solution of $Na_2S_2O_3$ and brine. The organic layer was dried over anhydrous Na_2SO_4 and concentrated under reduced pressure. The residue was purified by FC with CHX:AcOEt (99:1) as eluent to give the desired product. Yield: 74%, white solid, m.p.: 59-61 °C. ^{1}H NMR (300 MHz, $CDCl_3$) δ 8.10 (d, *J* = 8.8 Hz, 2H), 7.45 - 7.39 (m, 2H), 7.31 - 7.28 (m, 2H), 6.99 (d, *J* = 8.0 Hz, 2H), 3.84 (s, 3H). ^{13}C NMR in accordance with literature report. [2]

- **3-(3,5-dimethoxyphenyl)-2-(4-methoxyphenyl)benzofuran (16).**

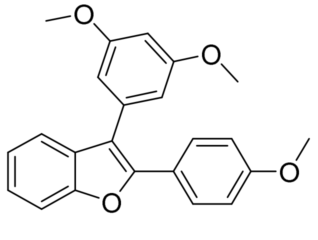

A mixture of **15** (0.04 mmol, 1 eq.), (3,5-dimethoxyphenyl)boronic acid (0.06 mmol, 1.4 eq.), K_2CO_3 (0.13 mmol, 3 eq.), $PdCl_2$(dppf), DCM (0.0021 mmol, 0.05 eq.) in THF:H_2O 1:1 previously degassed,

was heated in a MW vial at 70 °C for 30 min. After cooling down, the mixture was diluted with EtOAc and washed with H_2O. The aqueous phase was extracted with EtOAc. The combined organic phases was washed with brine, dried over anhydrous Na_2SO_4 and concentrated under reduced pressure. The residue was purified by FC with CHX:EtOAc (93:7) as eluent. Yield: 83%, light brown amorphous solid. ^{1}H NMR (300 MHz, $CDCl_3$) δ 7.69 – 7.62 (m, 2H), 7.56 – 7.50 (m, 2H), 7.34 – 7.28 (m, 1H), 7.26 – 7.20 (m, 1H), 6.91 – 6.84 (m, 2H), 6.66 (d, *J* = 2.3 Hz, 2H), 6.52 (t, *J* = 2.3 Hz, 1H), 3.83 (s, 3H), 3.79 (s, 6H).

- **5-(2-(4-hydroxyphenyl)benzofuran-3-yl)benzene-1,3-diol (3).**

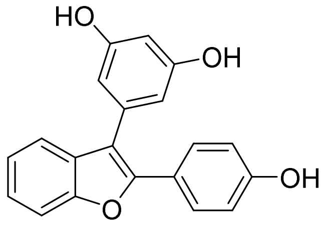

To a solution of **16** (0.11 mmol, 1 eq.) in dry DCM (1.3 mL), under N_2 at 0 °C a 1M BBr_3 solution in DCM (0.33 mmol, 2.9 eq.) was added dropwise. Then mixture was allowed to warm to room temperature. After 16 h the reaction solution was quenched with a cold saturated solution of $NaHCO_3$ (0 °C), concentrated under reduced pressure and diluted with H_2O. The aqueous phase was extracted with EtOAc for three times. The combined organic phases were dried over anhydrous Na_2SO_4 and concentrated under reduced pressure. The residue was purified by FC with DCM:MeOH (95:5) as eluent to give the desired product as a light brown amorphous solid. Yield: 61%. ^{1}H NMR: (300 MHz, CD_3OD) δ 7.56 – 7.51 (m, 2H), 7.50 – 7.41 (m, 2H), 7.31 – 7.16 (m, 2H), 6.79 – 6.73 (m, 2H), 6.40 (d, *J* = 2.2 Hz, 2H), 6.31 (t, *J* = 2.2 Hz, 1H). ^{13}C NMR (75 MHz, CD_3OD) δ 158.7 (×2), 157.7, 153.7, 150.7, 134.7, 130.2, 128.3 (×2), 123.7, 122.4, 121.9, 119.2, 115.4, 114.8 (×2), 110.2, 107.7 (×2), 101.5.

Synthesis of compound 4.

- **Methyl 2-(4-methoxyphenyl)benzofuran-5-carboxylate (18).**

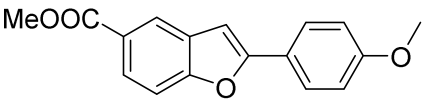

To a solution of methyl 4-hydroxy-3-iodobenzoate **17** (0.36 mmol, 1 eq.) in THF (0.95 mL) and TEA (1 mL, 7.19 mmol, 20 eq.), 1-ethynyl-4-methoxybenzene **13** (0.43 mmol, 1.2 eq.), CuI (0.01 mmol, 0.03 eq.) and $PdCl_2(PPh_3)_2$ (0.02 mmol, 0.05 eq.) were added under N_2 and the mixture was degassed and warmed to reflux (40 °C) under N_2 for 40 min. Then CH_3CN (1.9 mL) was added and the resulting mixture was warmed to 100 °C for 90 min, then it was allowed to cool at room temperature, washed twice with H_2O and concentred under reduced pressure. The residue was purified by FC with CHX:EtOAc (95:5) as eluent to give the desired product as a white sticky solid. Yield: 62%, ^{1}H NMR (300 MHz, $CDCl_3$) δ 8.28 (d, *J*=1.8 Hz, 1H), 7.98 (dd, *J*=8.6 Hz, 1.8 Hz, 1H), 7.80 (d, *J*=8.9 Hz, 2H), 7.51 (d, *J*=8.6 Hz, 1H), 6.99 (d, *J*= 8.9 Hz, 2H), 6.93 (d, *J*=0.8 Hz, 1H), 3.94 (s, 3H), 3.87 (s, 3H).

- **2-(4-methoxyphenyl)benzofuran-5-carbaldehyde (19).**

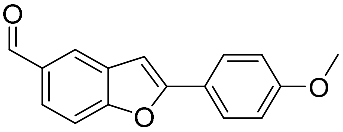

To the solution of compound **18** (0.99 mmol, 1 eq.) in THF dry (9.5 mL) under N_2 at 0 °C 1M $LiAlH_4$ in THF (2.97 mmol, 3 eq.) was added dropwise. The mixture was stirred for 10 min. at 0 °C, then it was quenched with HCl 1M at 0 °C. The aqueous phase was extracted with EtOAc for three times. The combined organic phases were dried by Na_2SO_4, filtered and concentrated under reduced pressure, then the residue (0.82 mmol, 1 eq.) was dissolved in in DCM (4.7 mL) and added with Dess-Martin periodinane (1.07 mmol, 1.3 eq.) at 0 °C. The mixture was stirred at room temperature for 2 h and the solvent was evaporated under reduced pressure. The residue was purified by FC with CHX:EtOAc (8:2) as eluent. Yield: 85%, white solid, m.p.: 121 °C. ^{1}H NMR (300 MHz, $(CD_3)_2CO$) δ 10.09 (s, 1H), 8.20 (dd, *J*=2.3 Hz, 0.5 Hz, 1H), 7.92 (d, *J*= 9.2 Hz, 2H), 7.89 (dd, *J*= 9.0, 2.0 Hz, 1H), 7.73 (d, *J*= 8.4 Hz, 1H), 7.32 (d, *J*= 0.8 Hz, 1H), 7.09 (d, *J*= 8.9 Hz, 2H), 3.88 (s, 3H).

- **(*E*)-5-(3,5-dimethoxystyryl)-2-(4-methoxyphenyl)benzofuran (10).**

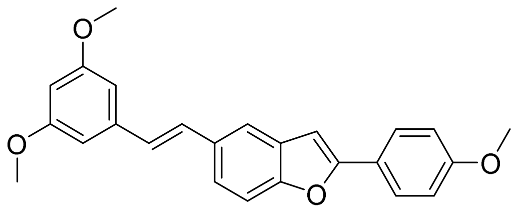

In a MW vial, compound **19** (0.55 mmol, 1 eq.) and diethyl (3,5-dimethoxybenzyl)phosphonate (0.83 mmol, 1.5 eq.) were solubilized in THF dry under N_2, then 60% NaH in mineral oil (1.66 mmol, 3 eq.) was added to the solution and the mixture was heated under microwave irradiation at 120 °C for 30 min. After cooling, the mixture was quenched by saturated solution od NH_4Cl and the aqueous phase was extracted with EtOAc for three times. The organic phases were washed with brine, dried with Na_2SO_4 and concentrated under reduced pressure. The crude product was purified by FC with CHX:EtOAc (8:2) as eluent. Yield: 76%, light yellow solid, m.p.: 169-171 °C. ^{1}H NMR (300 MHz, $CDCl_3$) δ 7.80 (d, *J*=8.8 Hz, 2H), 7.67 (s, 1H), 7.48 (d, *J*=8.4 Hz, 1H), 7.44 (dd, *J*=8.6 Hz, 1.6 Hz, 1H), 7.19 (d, *J*= 16.2 Hz, 1H), 7.03 (d, *J*= 15.7 Hz, 1H), 6.99 (d, *J*= 8.8 Hz, 2H), 6.88 (d, *J*= 0.5 Hz, 1H), 6.70 (d, *J*= 2.2 Hz, 2H), 6.40 (t, *J*= 2.2 Hz, 1H), 3.87 (s, 3H), 3.85 (s, 6H). ^{13}C NMR (75 MHz, $CDCl_3$) δ 161.0 (×2), 160.1, 156.8, 154.6, 139.7, 132.3, 130.0, 129.6, 127.5, 126.4 (×2), 123.2, 122.7, 118.6, 114.3 (×2), 111.1, 104.5 (×2), 99.7, 99.6, 55.3 (×3).

- **(*E*)-4-(5-(3,5-dimethoxystyryl)benzofuran-2-yl)phenol (9).**

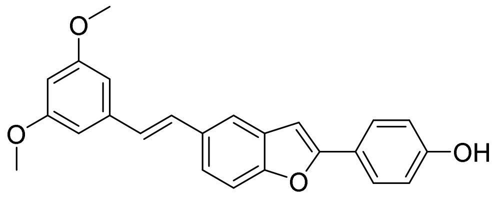

To a solution of **10** (0.12 mmol, 1 eq.) in dry DCM (1.3 mL), under N_2 at 0 °C a 1M BBr_3 solution in DCM (0.34 mmol, 2.9 eq.) was added dropwise, then the mixture was allowed to warm to room temperature. After 16 h the reaction was quenched with H_2O (0 °C) and concentrated under reduced pressure. The aqueous phase was extracted with EtOAc for three times. The combined organic phases were dried over anhydrous Na_2SO_4, filtered and concentrated under reduced pressure. The residue was purified by FC with CHX:EtOAc (8:2) as eluent to give the desired product. Yield: 60%, pale yellow solid, m.p.:194 – 195 °C.

^{1}H NMR: (300 MHz, $(CD_3)_2CO$) δ 7.80 (d, *J*= 9.0 Hz, 2H), 7.78 (s, 1H), 7.55 (dd, *J*= 2.3 Hz, 0.9 Hz, 1H), 7.51 (d, *J*= 2.3 Hz, 1H), 7.36 (d, *J*= 8.4 Hz, 1H), 7.17 (d, *J*= 15.0 Hz, 1H), 7.09 (d, *J*= 15.0 Hz, 1H), 6.98 (d, *J*=9.0 Hz, 2H), 6.80 (d, *J*= 0.9 Hz, 2H), 6.41 (t, *J*= 2.3 Hz, 1H) 3.83 (s, 6H). ^{13}C NMR (75 MHz, $(CD_3)_2CO$)

δ 161.2 (×2), 158.3, 157.1, 154.4, 139.8, 132.7, 130.2, 129.3, 127.5, 126.5 (×2), 122.7, 121.9, 118.6, 115.8 (×2), 110.8, 104.3 (×2), 99.5, 99.3, 54.7 (×2).

- **(*E*)-5-(2-(2-(4-hydroxyphenyl)benzofuran-5-yl)vinyl)benzene-1,3-diol (4).**

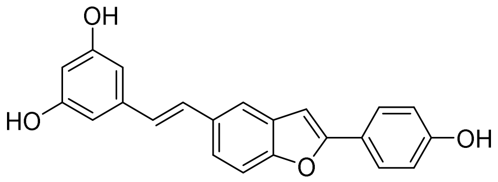

To a solution of compound **10** (0.08 mmol, 1 eq.) and TBAI (0.70 mmol, 9 eq.) in DCM dry (4.8 mL) 1M BCl_3 in DCM (0.70 mmol, 9 eq.) was added dropwise at 0 °C under N_2. The mixture was stirred at room temperature. After 6 h the reaction solution was quenched with H_2O at 0 °C and diluted with EtOAc. The organic phase was washed with a 10% Na_2SO_3 solution. The aqueous phase was extracted twice with EtOAc. The combined organic phases were washed again with brine, dried over anhydrous Na_2SO_4, filtered and concentrated under reduced pressure. The residue was purified by FC with CHX:Acetone (6:4) as eluent. The product was purified again with preparative HPLC-UV. Chromatographic separation was performed using Kromasil 5 – AmyCoat (250 × 21.2 mm, λ = 220 nm) and an isocratic elution (hexane: *i*PrOH 60:40, rate flow 15 mL/min). Product **4**, t_R = 6.75 min. Yield: 35%, white sticky solid. ^{1}H NMR: (300 MHz, CD_3OD) δ 7.80 (d, *J*= 9.0 Hz, 2H), 7.78 (s, 1H), 7.55 (dd, *J*= 2.3 Hz, 0.9 Hz, 1H), 7.51 (d, *J*= 2.3 Hz, 1H), 7.36 (d, *J*= 8.4 Hz, 1H), 7.17 (d, *J*= 15.0 Hz, 1H), 7.09 (d, *J*= 15.0 Hz, 1H), 6.98 (d, *J*=9.0 Hz, 2H), 6.80 (d, J=0.9 Hz, 2H), 6.41 (t, *J*= 2.3 Hz, 1H) 3.83 (s, 6H). ^{13}C NMR (75 MHz, CD_3OD) δ 158.3 (×2), 158.1, 157.1, 154.3, 139.7, 132.7, 130.1, 128.5, 127.4, 126.1 (×2), 122.1, 121.9, 118.0, 115.3 (×2), 110.4, 104.6 (×2), 101.5, 98.7.

Synthesis of compound 5.

- **Methyl 4-(2,2-dimethoxyethoxy)benzoate (21).**

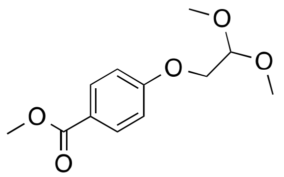

To a solution of methyl 4-hydroxybenzoate **20** (3.29 mmol, 1 eq.) in dry ACN under N_2 2-bromo-1,1-dimethoxyethane (4.98 mmol, 1.5 eq.) and Cs_2CO_3 (6.64 mmol, 2 eq.) were added. The mixture was stirred at reflux for three days. After cooling, the solvent was evaporated under reduced pressure, the crude was diluted in EtOAc and washed with H_2O. The aqueous phase was extracted twice with EtOAc. The combined organic phases were dried with Na_2SO_4 and concentrated under reduced pressure. The residue was purified by FC with CHX:DCM:EtOAc (10:2:1) as eluent. Yield: 61%, yellow oil. ^1H NMR (300 MHz, $CDCl_3$) δ 8.0 (d, *J* = 9.2 Hz, 2H), 6.94 (d, *J*=9.2 Hz, 2H), 4.73 (t, *J*= 5.2 Hz, 1H), 4.05 (d, *J*= 5.2 Hz, 2H), 3.88 (s, 3H), 3.46 (s, 6H). ^{13}C NMR (75 MHz, $CDCl_3$) δ 166.6, 162.1, 131.5 (×2), 122.9, 114.1 (×2), 101.9, 67.6, 54.2 (×2), 51.8.

- **Methyl benzofuran-5-carboxylate (22).**

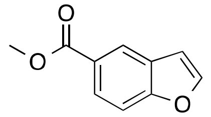

To a solution of compound **21** (1.55 mmol, 1 eq.) in toluene (22.8 mL) Amberlyst-15 (10 wt%) was added The mixture was heated at reflux for 6 h. After cooling, the mixture was filtered, and the solvent was evaporated under reduced pressure. The residue was purified by FC with CHX:EtOAc (8:2) as eluent. Yield: 51%, white solid.^1H NMR (300 MHz, $CDCl_3$) δ 8.35 (d, *J* = 1.8 Hz, 1H), 8.03 (dd, *J*= 8.7, 1.8 Hz, 1H), 7.69 (d, *J*= 2.2 Hz, 1H), 7.53 (dd, *J*= 8.7, 0.8 Hz, 1H), 6.84 (dd, J= 2.2, 0.8 Hz, 1H), 3.89 (s, 3H).

- **Methyl 2,3-dibromo-2,3-dihydrobenzofuran-5-carboxylate (23).**

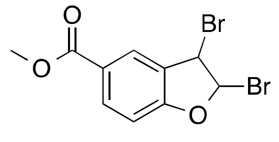

To a solution of compound **22** (0.79 mmol, 1 eq.) in dry DCM (0.6 mL) under N_2 at 0 °C Br_2 (0.79 mmol, 1 eq.) was added dropwise and the mixture was stirred at r.t. for 75 min. The solution was quenched with a 1M Na_2SO_3 solution. The aqueous phase was extracted with EtOAc for three times. The

combined organic phases were washed with brine, dried with Na_2SO_4, filtered and concentrated under reduced pressure. The residue was purified by FC with CHX:EtOAc (95:5) as eluent. Yield: 82%, white sticky solid. ^{1}H NMR (300 MHz, $CDCl_3$) δ 8.23 (d, *J*=1.9 Hz, 1H), 8.11 (dd, *J*= 8.6 Hz, 1.9 Hz, 1H), 7.10 (d, *J*= 8.6 Hz, 1H), 6.93 (s, 1H), 5.75 (s, 1H), 3.92 (s, 3H). ^{13}C NMR (75 MHz, $CDCl_3$) δ 165.8, 160.3, 133.7, 127.8, 126.8, 126.6, 112.3, 90.2, 52.3, 51.6.

- **Methyl 3-bromobenzofuran-5-carboxylate (24).**

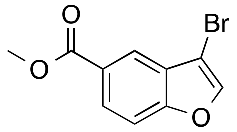

To a solution of compound **23** (0.56 mmol, 1 eq.) in dry THF (0.76 mL) at 0 °C KOH 85% (0.56 mmol, 1 eq.) and MeOH (152 μL) were added. The mixture was stirred for 20 min., after that it was diluted with EtOAc and washed with H_2O. The aqueous phase was extracted twice with EtOAc. The combined organic phases were washed with a sat. $NaHCO_3$ solution, dried with Na_2SO_4, filtered and concentrated under reduced pressure. The residue was purified by FC with CHX:EtOAc (95:5) as eluent. Yield: 82%, white sticky solid. ^{1}H NMR (300 MHz, $CDCl_3$) δ 8.30 (d, *J*=1.8 Hz, 1H), 8.09 (dd, *J*=8.8 Hz, 1.8 Hz, 1H), 7.72 (s, 1H), 7.53 (d, *J*=8.8 Hz, 1H), 7.26 (s, 1H), 3.97 (s, 3H). ^{13}C NMR (75 MHz, $CDCl_3$) δ 166.7, 156.8, 143.9, 143.8, 127.1, 125.9, 122.3, 111.7, 98.4, 52.2.

- **Methyl 3-(3,5-dimethoxyphenyl)benzofuran-5-carboxylate (25).**

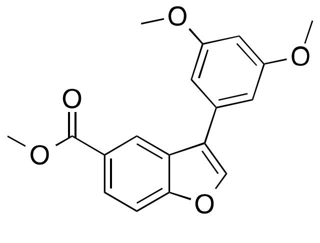

A solution of compound **24** (0.45 mmol, 1 eq.), 3,5-dimethoxyphenylboronic acid (0.50 mmol, 1.1 eq.) and Na_2CO_3 (1.0 mmol, 2.2 eq.) in DME:H_2O 5:1 was degassed for 30 min. Then, $Pd(PPh_3)_4$ (0.01 mmol, 0.03 eq.) was added and the mixture was degassed again. The reaction was heated at 80 °C overnight. After cooling, the mixture was quenched with water and the aqueous phase was extracted three times with EtOAc. The organic phases were washed with brine, dried by Na_2SO_4 and concentrated. The crude was purified on FC with CHX:EtOAc 9:1 as eluent. Yield: 74%, white solid, m.p. = 101-103 °C. ^{1}H NMR (300 MHz, $CDCl_3$) δ 8.56 (d, *J*= 1.7 Hz, 1H), 8.09 (dd, *J*= 8.7 Hz, 1.7 Hz, 1H), 7.84 (s, 1H), 7.57 (d, *J*= 8.7 Hz, 1H), 6.78 (d, *J*= 2.3 Hz, 2H), 6.52 (t, *J*= 2.3 Hz, 1H) 3.96 (s, 3H), 3.87 (s, 6H). ^{13}C NMR (75 MHz, $CDCl_3$): δ 166.2, 160.2, 159.3 (×2), 143.0, 141.7, 136.2, 132.3, 130.9, 129.4, 128.7, 112.1, 104.5 (×2), 99.9, 55.8 (×2), 51.7.

- **3-(3,5-dimethoxyphenyl)benzofuran-5-carbaldehyde (26)**

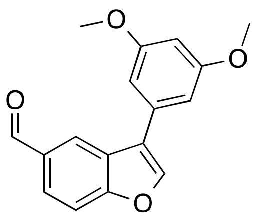

To a solution of compound **25** (0.23 mmol, 1 eq.) in THF dry under N_2 at 0°C was added $LiAlH_4$ 1M in THF (0.70 mmol, 3 eq.) dropwise. The mixture was stirred for 10 minutes at 0 °C, then it was quenched with HCl 1M at 0°C. The aqueous phase was extracted with EtOAc for three times. The organic phases were joined, dried by Na_2SO_4, filtered and concentrated under reduced pressure. Purification by FC using CHX:EtOAc (6:4) as eluent gave (3-(3,5-dimethoxyphenyl)benzofuran-5-yl)methanol as a brown sticky solid. Yield: 97%, ^{1}H NMR (300 MHz, $CDCl_3$) δ 7.83 (d, *J*= 1.1 Hz, 1H), 7.80 (s, 1H), 7.53 (d, *J*= 8.5 Hz, 1H), 7.37 (dd, *J*= 8.5, 1.7 Hz, 1H), 6.78 (d, *J*= 2.3 Hz, 2H), 6.50 (t, *J*= 2.3 Hz, 1H), 4.80 (s, 2H), 3.86 (s, 6H). ^{13}C NMR (75 MHz, $CDCl_3$) δ 161.2 (×2), 155.3, 142.0, 136.0, 133.7, 126.5, 124.2, 122.3, 119.1, 111.8, 105.8 (×2), 99.3, 65.6, 55.4 (×2).

To a solution of the above compound (0.21 mmol, 1 eq.) in DCM Dess-Martin periodinane (0.27 mmol, 1.3 eq.) was added at 0 °C. The mixture was stirred at room temperature for 90 min. and the solvent was evaporated under reduced pressure. The residue was purified by FC with CHX:EtOAc as eluent (8:2). Yield: 78%, yellow solid, m.p.: 96 - 98 °C. ^{1}H NMR (300 MHz, $CDCl_3$) δ 10.09 (s, 1H), 8.36 (d, *J*= 1.6 Hz, 1H), 7.93 (dd, *J*= 8.6, 1.6 Hz, 1H), 7.88 (s, 1H), 7.66 (d, *J*= 8.6 Hz, 1H), 6.53 (t, *J*= 2.3 Hz, 1H), 3.87 (s, 6H). ^{13}C NMR (75 MHz, $CDCl_3$) δ 191.6, 161.4 (×2), 159.0, 143.0, 132.7, 132.4, 127.1, 125.9, 124.1, 123.0, 112.6, 105.9 (×2), 99.7, 55.5 (×2).

- **(*E*)-3-(3,5-dimethoxyphenyl)-5-(3,5-dimethoxystyryl)benzofuran (27).**

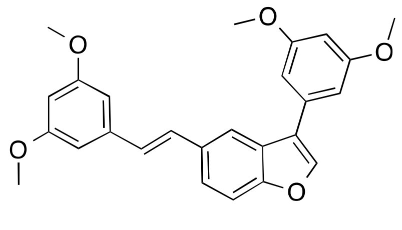

In a MW vial, compound **26** (0.15 mmol, 1 eq.) and diethyl (3,5-dimethoxybenzyl)phosphonate (0.22 mmol, 1.5 eq.) were solubilized in dry THF (2 mL) under N_2, then 60% NaH in mineral oil (0.45 mmol, 3 eq.) was added to the solution and the mixture was heated under microwave irradiation at 120 °C for 30 min. After cooling, the mixture was quenched by a saturated solution of NH_4Cl and the aqueous phase was extracted with EtOAc for three times. The organic phases were washed with brine, dried with Na_2SO_4 and concentrated under reduced pressure. Purification by FC with CHX:EtOAc (8:2) as eluent gave the title compound as a white sticky solid. Yield: 80%. ^{1}H NMR (300 MHz, $CDCl_3$) δ 7.85

(d, *J*= 1.5 Hz, 1H), 7.77 (s, 1H), 7.53 (dd, *J*= 8.6, 1.5 Hz, 1H), 7.49 (d, *J*= 8.6 Hz, 1H), 6.92 (d, *J*= 16.3 Hz, 1H), 6.89 (d, *J*= 16.3 Hz, 1H), 6.65 (d, *J*= 2.2 Hz, 2H), 6.51 (d, *J*= 2.2 Hz, 2H), 6.40 (t, *J*= 2.2 Hz, 1H), 6.35 (t, *J* = 2.2 Hz, 1H), 3.87 (s, 6H), 3.82 (s, 6H). ^{13}C NMR (75 MHz, $CDCl_3$) δ 161.3 (×2), 161.0 (×2), 155.5, 142.1, 139.5, 132.6, 129.5, 127.9, 126.9, 123.2, 122.4, 118.8, 111.9, 110.0, 105.4 (×2), 104.3 (×2), 99.9, 99.3, 55.5 (×2), 55.4 (×2).

- **(*E*)-5-(2-(3-(3,5-dihydroxyphenyl)benzofuran-5-yl)vinyl)benzene-1,3-diol (5).**

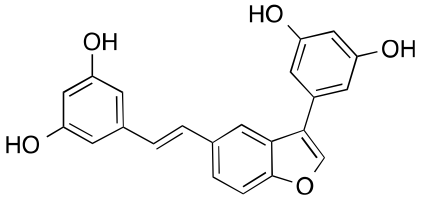

To a solution of compound **27** (0.11 mmol, 1 eq.) in dry DCM (7 mL) under N_2 a 1M BBr_3 solution in DCM (1.37 mmol, 12 eq.) was added dropwise at 0 °C. The mixture was stirred at r.t. for 9 h. The reaction was quenched with H_2O at 0 °C. The aqueous phase was extracted with EtOAc for three times. The combined organic phases were dried over anhydrous Na_2SO_4 and concentrated under reduced pressure. The residue was purified by FC with DCM:MeOH (95:5) to give the desired product. Yield: 14%, light brown sticky solid. ^{1}H NMR (300 MHz, CD_3OD) δ 7.93 (d, *J*= 1.5 Hz, 1H), 7.90 (s, 1H), 7.57 (dd, *J*= 8.6, 1.5 Hz, 1H), 7.49 (d, *J*= 8.6 Hz, 1H), 7.19 (d, *J*= 16.3 Hz, 1H), 7.00 (d, *J*= 16.3 Hz, 1H), 6.65 (d, *J* = 2.2 Hz, 2H), 6.51 (d, *J*= 2.2 Hz, 2H), 6.31 (t, *J*= 2.2 Hz, 1H), 6.19 (t, *J*= 2.2 Hz, 1H). ^{13}C NMR (150 MHz, CD_3OD) δ 160.1 (×2), 159.7 (×2), 156.9, 143.4, 140.9, 134.8, 134.2, 129.7, 129.2, 128.0, 124.2, 123.6, 119.5, 112.7, 106.9 (×2), 106.0 (×2), 103.0, 102.8.

Synthesis of compounds 6 and 7.

- ***2-bromo-1-(3,5-dimethoxyphenyl)ethan-1-one* (29).**

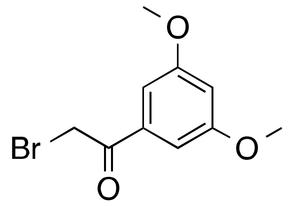

To a solution of 1-(3,5-dimethoxyphenyl)ethan-1-one **28** (11.09 mmol, 1 eq) in a mixture of EtOAc:$CHCl_3$ (1:1, v/v, 30 mL) $CuBr_2$ (22.19 mmol, 2 eq) was added. The mixture was stirred at reflux overnight and it was cooled and filtered on a celite pad using EtOAc as eluent. The solvent was evaporated under reduced pressure. The crude was purified by FC with CHX:DCM:EtOAc 10:1:0.5 as eluent. Yield: 67%; orange amorphous solid. ^1H NMR (300 MHz, $CDCl_3$) δ (ppm): 7.11 (d, 2H, *J*=2.3 Hz), 6.69 (t, 1H, *J*=2.3 Hz), 4.42 (s, 2H), 3.84 (s, 6H). ^{13}C NMR in accordance to literature.[3]

- **1-(3,5-dimethoxyphenyl)-2-(3-methoxyphenoxy)ethan-1-one (30).**

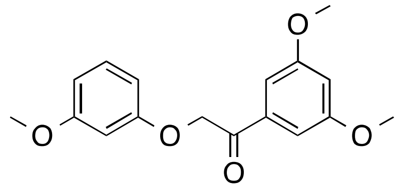

A solution of **29** (1.15 mmol), 3-methoxyphenol (1.23 mmol, 1.1 eq) and K_2CO_3 (3.47 mmol, 3 eq) in dry acetone (3.2 mL) under nitrogen was heated to reflux. After 2h, it was cooled to r.t., concentrated under reduced pressure, diluted with EtOAc and washed with H_2O. The aqueous phase was extracted twice with EtOAc. The combined organic phases were washed with H_2O, dried over anhydrous Na_2SO_4 and concentrated under reduced pressure. The residue was purified by FC with CHX:AcOEt:DCM (10:1:2) as eluent. Yield: 90%, yellow-orange sticky solid. ^1H NMR (300 MHz, $CDCl_3$) δ 7.22 – 7.14 (m, 1H), 7.12 (d, *J*= 2.3 Hz 2H), 6.69 (t, *J*= 2.3 Hz, 1H), 6.58 – 6.49 (m, 3H), 5.23 (s, 2H), 3.84 (s, 6H), 3.78 (s, 3H).

- **3-(3,5-dimethoxyphenyl)-6-methoxybenzofuran (31).**

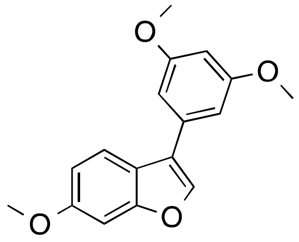

To a solution of compound **30** (0.33 mmol, 1 eq.) in DCM (3.9 mL) under nitrogen atmosphere $Bi(OTf)_3$ (0.07 mmol, 0.2 eq.) was added. The mixture was stirred at reflux overnight. After cooling the mixture was filtered on a celite pad washing with DCM and then concentrated under reduced pressure. The crude was purified by FC (CHX:EtOAc from 9:1 to 7:3). Yield: 43%, white solid, m.p. 87 °C. ^{1}H NMR (300 MHz, $CDCl_3$) δ 7.72 (s, 1H), 7.70 (d, *J*= 8.6 Hz, 1H), 7.07 (d, *J*= 2.3 Hz, 1H), 6.95 (dd, *J*= 8.6, 2.3 Hz, 1H), 6.78 (d, *J*= 2.3 Hz, 2H), 6.49 (t, *J*= 2.3 Hz, 1H), 3.88 (s, 3H), 3.86 (s, 6H). ^{13}C NMR (75 MHz, $CDCl_3$) δ 161.2 (×2), 158.2, 156.8, 140.6, 134.0, 122.2, 120.6, 119.7, 112.1, 105.6 (×2), 99.4, 96.2, 55.7, 55.4 (×2).

- **3-(3,5-dimethoxyphenyl)-6-methoxy-2-(4-methoxyphenyl)benzofuran (11).**

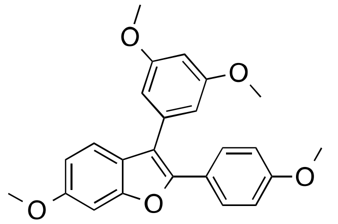

A mixture of compound **31** (0.14 mmol, 1eq.), $Pd(OAc)_2$ (0.01 mmol, 0.1 eq.), 4-bromoanisole (0.28 mmol, 2 eq.), $PCy_3{\cdot}HBF_4$ (0.03 mmol, 0.2 eq.), K_2CO_3 (0.21 mmol, 1.5 eq.) and pivalic acid (0.42 mmol, 3 eq.) in DMA (0.6 mL) was degassed. Then, the reaction mixture was heated at 100 °C under stirring for 20 h. After cooling, the mixture was diluted with EtOAc, washed with H_2O and saturated solution $NaHCO_3$. The organic phases were dried over anhydrous Na_2SO_4 and concentrated under reduced pressure. FC was used to purified the crude with CHX:AcOEt:DCM from 10:0.1:1 to 9:1:2 as eluent. Yield: 80%, orange sticky solid. ^{1}H NMR (300 MHz, $CDCl_3$) δ 7.64 – 7.57 (m, 2H), 7.38 (d, *J*= 8.6 Hz, 1H), 7.08 (d, *J*= 2.2 Hz, 1H), 6.89 – 6.83 (m, 3H), 6.64 (d, *J*= 2.3 Hz, 2H), 6.50 (t, *J*= 2.3 Hz, 1H), 3.89 (s, 3H), 3.82 (s, 3H), 3.78 (s, 6H). ^{13}C NMR (75 MHz, $CDCl_3$) δ 161.2 (×2), 159.5, 158.1, 154.7, 149.9, 135.1, 128.2 (×2), 123.7, 123.5, 120.0, 115.8, 113.8 (×2), 111.7, 107.6 (×2), 99.8, 95.7, 55.8, 55.4 (×2), 55.3.

- **2-(3-bromo-5-methoxyphenoxy)-1-(3,5-dimethoxyphenyl)ethan-1-one (32).**

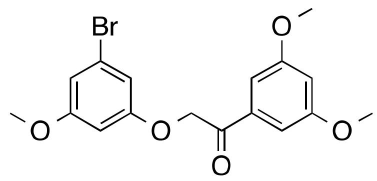

A mixture of compound **29** (2.46 mmol, 1 eq.), 3-bromo-5-methoxyphenol (2.46 mmol, 1 eq.) and K_2CO_3 (7.39 mmol, 3 eq) in dry acetone (7 mL) was stirred at reflux for 2h. After this time the mixture was cooled to room temperature, concentrated under reduced pressure, diluted with EtOAc and washed with H_2O. The aqueous phase was extracted again with EtOAc. The collected organic phases dried over anhydrous Na_2SO_4, filtered and concentrated. The crude was purified by FC (CHX:EtOAc from 9:1 to 8:2). Yellow solid, yield: 89%, m.p.:106-108 °C. ^{1}H NMR (300 MHz, $CDCl_3$) δ 7.10 (d, *J*= 2.3 Hz,

2H), 6.70 (t, *J*= 2.1 Hz, 2H), 6.67 (dd, *J*= 2.3, 1.6 Hz, 1H), 6.45 (t, *J*= 2.3 Hz, 1H), 5.21 (s, 2H), 3.85 (s, 6H), 3.76 (s, 3H). ^{13}C NMR in accordance to literature.[3]

- **4-bromo-3-(3,5-dimethoxyphenyl)-6-methoxybenzofuran (33).**

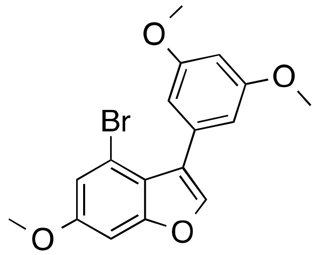

To a solution of compound **32** (0.33 mmol, 1 eq) in DCM (3.9 mL) under nitrogen atmosphere $Bi(OTf)_3$ (0.07 mmol,0.2 eq.) was added. The mixture was stirred at reflux overnight. After cooling, the mixture was filtered on a celite pad washing with DCM and then concentrated under reduced pressure. The crude was purified by FC (CHX:EtOAc from 9:1to 7:3). Yield: 83%, white sticky solid.^{1}H NMR (300MHz, $CDCl_3$) δ 7.54 (s, 1H), 7.10 (d, *J*= 2.2 Hz, 1H), 7.03 (d, *J*= 2.2 Hz, 1H), 6.66 (d, *J*= 2.3 Hz, 2H), 6.51 (t, *J*= 2.3 Hz, 1H), 3.86 (s, 3H), 3.83 (s, 6H). ^{13}C NMR in accordance to literature.[3]

- **5-(4-bromo-6-hydroxybenzofuran-3-yl)benzene-1,3-diol (34).**

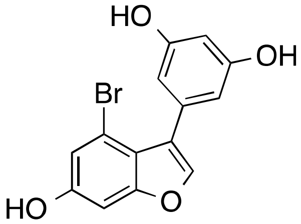

To a solution of **33** (0.14 mmol, 1 eq.) in dry DCM (1.45 mL), under nitrogen and at 0 °C, a 1M BBr_3, solution in DCM, (0.40 mmol, 3 eq.) was added dropwise. The reaction mixture was allowed to warm to room temperature. After 16 h, the reaction mixture was quenched with a cold saturated solution of $NaHCO_3$ (0°C), concentrated under reduced pressure, diluted with H_2O. The aqueous phase was extracted with EtOAc for three times. The combined organic phases were dried over anhydrous Na_2SO_4 and concentrated under reduced pressure. The residue was purified by FC with DCM:MeOH (95:5) as eluent to give the desired product. Yield: 91%, light brown oil.^{1}H NMR (300 MHz, CD_3OD) δ 7.54 (s, 1H), 6.95 (d, *J*= 2.0 Hz, 1H), 6.90 (d, *J*= 2.0 Hz, 1H), 6.39 (d, *J*= 2.2 Hz, 2H), 6.29 (t, *J*= 2.2 Hz, 1H). ^{13}C NMR in accordance to literature.[3]

- **5-(6 hydroxy-2-(4-hydroxyphenyl)benzofuran-3-yl)benzene-1,3-diol (6).**

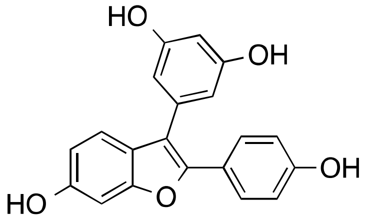

To a solution of **11** (0.12 mmol, 1 eq.) in dry DCM (1.2 mL), under nitrogen and at 0 °C, a 1M BBr_3 solution in DCM (0.45 mmol, 3.9 eq.) was added dropwise. The reaction mixture was allowed to warm to room temperature. After 16 h, the reaction mixture was quenched with a cold saturated solution of $NaHCO_3$ (0°C), concentrated under reduced pressure, diluted with H_2O. The aqueous phase was extracted with EtOAc for three times. The combined organic phases were dried over anhydrous Na_2SO_4 and concentrated under reduced pressure. The residue was purified by FC CHX:acetone (6:4) to give the desired product. Yield: 52%, light brown solid, m.p.: 239-240°C. ^{1}H NMR (300 MHz, CD_3OD) δ 7.50 – 7.43 (m, 2H), 7.24 (d, *J*= 8.6 Hz, 1H), 6.91 (d, *J*= 2.2 Hz, 1H), 6.77 – 6.70 (m, 3H), 6.38 (d, *J*= 2.2 Hz, 2H), 6.29 (t, *J*= 2.2 Hz, 1H). ^{13}C NMR (75 MHz, CD_3OD) δ 158.6 (×2), 157.2, 155.3, 154.7, 149.3, 135.1, 127.8 (×2), 122.8, 122.3, 119.4, 115.4, 114.8 (×2), 111.5, 107.7 (×2), 101.3, 96.9.

- **(E)-5-(6-hydroxy-4-(4-hydroxystyryl)benzofuran-3-yl)benzene-1,3-diol (7).**

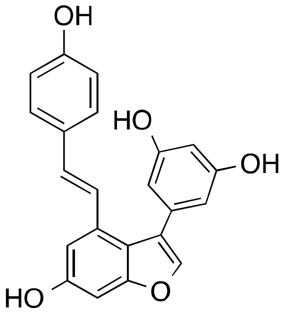

A mixture of compound **34** (0.45 mmol, 1eq.), 4-hydroxystirene (0.78 mmol, 1.5 eq.), dppp (0.05 mmol, 0.1 eq.) and TEA (0.91 mmol, 2 eq.) in DMF (14.6 mL) was degassed. $Pd(OAc)_2$ (0.05 mmol, 0.1 eq.) was added to the mixture, then the reaction mixture was heated at 120 °C under stirring for 20 h. After cooling, DMF was evaporated, the mixture was diluted with EtOAc, washed with H_2O and brine. The collected organic phases were dried with Na_2SO_4 and concentrated under reduced pressure. FC was used to purify the crude with CHX:Acetone 6:4 as eluent. Yield: 80%, light brown solid, m.p.:234-235°C. ^{1}H-NMR (300 MHz, CD_3OD) δ 7.50 (s, 1H), 7.20 (d, *J*= 16.3 Hz, 1H), 7.11 – 7.06 (m, 2H), 7.05 (d, *J*= 2.0 Hz, 1H), 6.91 (d, *J*= 16.3 Hz, 1H), 6.79 (d, *J*= 2.0 Hz, 1H), 6.70 – 6.64 (m, 2H), 6.43 (d, *J*= 2.2 Hz, 2H), 6.38 (t, *J*= 2.2 Hz, 1H). ^{13}C NMR (75 MHz, CD_3OD) δ 158.2 (×2), 156.9, 155.4, 140.4, 135.1, 132.2, 129.2, 128.4, 127.4 (×2), 123.2, 122.4, 117.8, 114.9 (×2), 110.0, 108.4 (×2), 106.5, 101.4, 96.4.

Synthesis of compound 8.

- **Methyl 3-hydroxy-5-methoxybenzoate (36).**

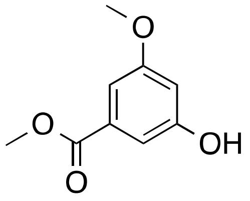

To a solution of 3,5-hydroxymethoxybenzoate **35** (5.94 mmol, 1 eq.) in dry DMF (7.5 mL) under N_2 methyl iodide (5.94 mmol, 1 eq.) and K_2CO_3 (8.92 mmol, 1.5 eq.) were added. The mixture was stirred at room temperature for 2 days. Then, the reaction was quenched with saturated solution NH_4Cl and the mixture was extracted with EtOAc for three times. The collected organic phases were dried with Na_2SO_4 and concentrated under reduced pressure. FC was used to purify the crude with CHX:EtOAc 7:3 as eluent. Yield: 35%, white sticky solid.

^{1}H NMR (300 MHz, $CDCl_3$) δ 7.17 - 7.15 (m, 2H), 6.63 (t, *J*= 2.4 Hz, 1H), 5.52 (s, 1H), 3.91 (s, 3H), 3.82 (s, 3H). [4]

- **Methyl 3-(2,2-dimethoxyethoxy)-5-methoxybenzoate (37).**

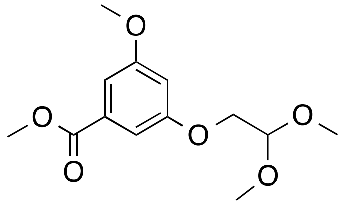

To a solution of compound **36** (2.74 mmol, 1 eq.) in ACN dry under N_2 2-bromo-1,1-dimethoxyethane (4.18 mmol, 1.5 eq.) was added dropwise, followed by Cs_2CO_3 (5.54 mmol, 2 eq.). The mixture was stirred at 85 °C for three days. After cooling, the solvent was evaporated under reduced pressure, the crude was diluted in EtOAc and washed with H_2O. The aqueous phase was extracted with EtOAc and the collected organic phases were dried with Na_2SO_4 and concentrated under reduced pressure. The residue was purified by FC with CHX:DCM:EtOAc (10:2:1) as eluent. Yield: 67%, yellow oil. ^{1}H NMR (300 MHz, $CDCl_3$) δ 7.20 (d, *J*= 2.3 Hz, 2H), 6.69 (t, *J*= 2.3 Hz, 1H), 4.72 (t, *J*= 5.2 Hz, 1H), 4.03 (d, *J*= 5.1 Hz, 3H), 3.90 (s, 3H), 3.82 (s, 3H), 3.46 (s, 6H). ^{13}C NMR in accordance to literature. [5]

- **Methyl 6-methoxybenzofuran-4-carboxylate (38).**

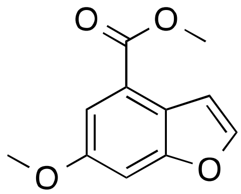

To a solution of compound **37** (0.37 mmol, 1 eq.) in chlorobenzene (3 mL) Amberlyst-15 (10 wt%) was added. The mixture was heated at 120 °C for 4 h. After cooling, the mixture was filtered, and the solvent was evaporated under reduced pressure. The residue was purified by FC with CHX:EtOAc 9:1 as eluent. Yield: 63%, sticky white solid. ^{1}H NMR (300 MHz, $CDCl_3$) δ 7.63 (d, *J*= 2.2 Hz, 1H), 7.60 (d, *J*= 2.2 Hz, 1H), 7.26 – 7.20 (m, 2H), 3.98 (s, 3H), 3.89 (s, 3H). ^{13}C NMR in accordance with literature. [5]

- **Methyl 2-bromo-6-methoxybenzofuran-4-carboxylate (39).**

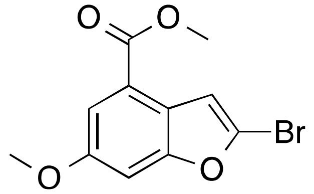

To a solution of compound **38** (0.23 mmol, 1 eq.) in 1,2 dichloroethane NBS (0.35 mmol, 1.5 eq) and a catalytic quantity of DMF (12 μL) were added. The mixture was stirred at 70 °C for 4h. To quench the reaction, a saturated solution of $Na_2S_2O_3$ was added to the mixture and the aqueous phase was extracted with EtOAc for three times. The organic phases were joined, dried over anhydrous Na_2SO_4 and concentrated under reduced pressure. The crude was purified by FC with CHX:EtOAc 95:5 as eluent. Yield: 80%, white sticky solid. ^{1}H NMR (300 MHz, $CDCl_3$) δ 7.57 (d, *J*= 2.3 Hz, 1H), 7.22 (d, *J*= 0.9 Hz, 1H), 7.18 (dd, *J*= 2.3, 0.9 Hz, 1H), 3.97 (s, 3H), 3.88 (s, 3H). ^{13}C NMR in accordance with literature. [5]

- **Methyl 6-methoxy-2-(4-methoxyphenyl)benzofuran-4-carboxylate (40).**

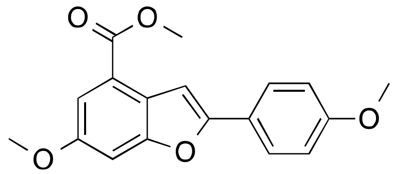

A solution of compound **39** (0.17 mmol, 1 eq.), 4-methoxyphenylboronic acid (0.35 mmol, 2 eq.), K_2CO_3 (0.92 mmol, 5.3 eq.) was degassed for 30 min. Then, $Pd(PPh_3)_4$ (0.005 mmol, 0.03 eq.) was added and the mixture was degassed again. The reaction was heated at 70 °C overnight. After cooling, the mixture was quenched with water and the aqueous phase was extracted three times with EtOAc. The organic phases were washed with brine for one time, dried by Na_2SO_4 and concentrated. The crude was purified on FC with CHX:EtOAc (9:1) as eluent. Yield: 91%, white solid. m.p. = 101-103 °C. ^{1}H NMR (300 MHz, $CDCl_3$) δ 7.79 (d, *J*= 9.0 Hz, 2H), 7.56 (d, *J*= 2.3 Hz, 1H), 7.39 (d, *J*= 0.9 Hz, 1H), 7.25 (dd, *J*= 2.3 Hz, 0.9 Hz, 1H), 6.97 (d, *J*= 9.0 Hz, 2H), 4.00 (s, 3H), 3.90 (s, 3H), 3.87 (s, 3H). ^{13}C NMR (75 MHz, $CDCl_3$): ^{13}C NMR in accordance with literature. [5]

- **6-methoxy-2-(4-methoxyphenyl)benzofuran-4-carbaldehyde (41).**

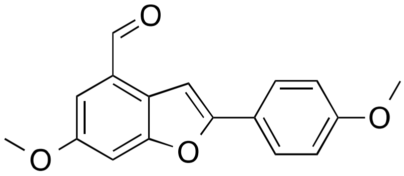

To the solution of compound **40** (0.38 mmol, 1 eq.) in THF dry under N_2 at 0 °C was added $LiAlH_4$ 1M in THF (1.06 mmol, 3 eq.) dropwise. The mixture was stirred for 10 minutes at 0 °C, then it was quenched with HCl 1M at 0 °C. The aqueous phase was extracted with EtOAc for three times. The organic phases were joined, dried by Na_2SO_4, filtered and concentrated under reduced pressure. Purification by FC with CHX:EtOAc (7:3) as eluent gave (6-methoxy-2-(4-methoxyphenyl)benzofuran-4-yl)methanol as a yellow solid. Yield: 89%. m.p.: 98-99 °C. ^1H NMR (300 MHz, $CDCl_3$) δ 7.74 (d, *J*= 9.0 Hz, 2H), 6.99 – 6.93 (m, 4H), 6.86 (d, *J*= 0.9 Hz, 1H), 4.88 (s, 2H), 3.86 (s, 3H), 3.85 (s, 3H). ^{13}C NMR (75 MHz, $CDCl_3$) δ 159.7, 157.7, 155.8, 155.7, 133.5, 126.0 (×2), 123.6, 121.3, 114.3 (×2), 110.0, 98.1, 95.3, 63.6, 55.9, 55.4.

To the solution of the above compound (0.27 mmol, 1 eq.) in DCM Dess-Martin periodinane (0.35 mmol, 1.3 eq.) was added at 0 °C. The mixture was stirred at room temperature for 75 min.and then the solvent was evaporated under reduced pressure. The residue was purified by FC with CHX:EtOAc as eluent (9:1 to 7:3). Yield: 97%, yellow solid, m.p.: 121 °C. ^1H NMR (300 MHz, $CDCl_3$) δ 10.14 (s, 1H), 7.80 (d, *J*= 9.0 Hz, 2H), 7.52 (d, *J*= 0.9 Hz, 1H), 7.30 – 7.28 (m, 2H), 6.98 (d, *J*= 9.0 Hz, 1H), 3.92 (s, 3H), 3.86 (s, 3H). ^{13}C NMR (75 MHz, $CDCl_3$) δ 191.6, 160.2, 158.3, 157.1, 156.1, 128.2, 126.4 (×2), 122.7, 122.5, 115.3, 114.3 (×2), 102.2, 99.3, 56.0, 55.4.

- **(*E*)-6-methoxy-2-(4-methoxyphenyl)-4-(4-methoxystyryl)benzofuran (42).**

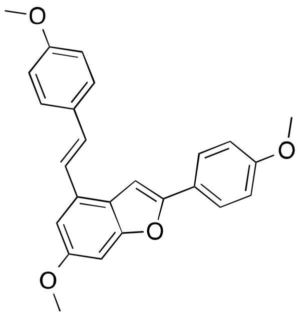

In a MW vial, compound **41** (0.23 mmol, 1 eq.) and diethyl (4-methoxybenzyl)phosphonate (0.46 mmol, 2 eq.) were solubilized in THF dry under N_2, then 60% NaH in mineral oil (0.69 mmol, 3 eq.) was added to the solution and the mixture was heated under microwave irradiation at 120 °C for 30 min. After cooling, the mixture was quenched by saturated solution of NH_4Cl and the aqueous phase was extracted with EtOAc for three times. The organic phases were washed with brine, dried with

Na_2SO_4 and concentrated under reduced pressure. Purification of the crude by FC with CHX:EtOAc (8:2) as eluent gave the title compound. Yield: 54%, light yellow oil. ^1H NMR (300 MHz, $CDCl_3$) δ 7.78 (d, *J*= 9.0 Hz, 2H), 7.52 (d, *J*= 9.0 Hz, 2H), 7.20 (d, *J*= 2.3 Hz, 2H), 7.08 (d, *J*= 0.9, 1H), 7.04 (d, *J*= 2.3 Hz, 1H), 6.99 – 6.92 (m, 5H), 3.90 (s, 3H), 3.86 (s, 3H), 3.85 (s, 3H). ^{13}C NMR (75 MHz, $CDCl_3$) δ 159.7, 159.6, 157.7, 156.0, 155.3, 130.6, 130.2, 129.9, 127.8, 126.0 (×2), 124.0, 123.5, 121.6, 114.3 (×2), 114.2, 113.7, 110.0, 107.9, 98.4, 95.2, 55.8, 55.3 (×2).

- **(*E*)-2-(4-hydroxyphenyl)-4-(4-hydroxystyryl)benzofuran-6-ol (8).**

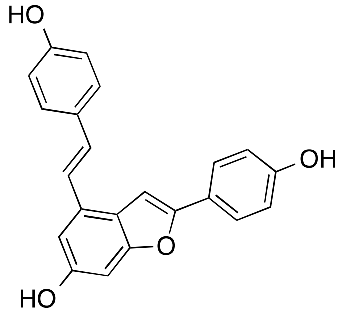

To a solution of compound **42** (0.07 mmol, 1 eq.) a 1M solution of BBr_3 in DCM (0.65 mmol, 9 eq.) was added dropwise at 0 °C. The mixture was stirred at rt for 6 h. The reaction was quenched with H_2O at 0 °C. The aqueous phase was extracted with EtOAc for three times. The combined organic phases were dried over anhydrous Na_2SO_4 and concentrated under reduced pressure. The residue was purified by FC (DCM/MeOH 95:5) to give the desired product. Yield: 24%, brown sticky solid. ^1H NMR (300 MHz, $CDCl_3$) δ 7.70 (d, *J*= 9.0 Hz, 2H), 7.47 (d, *J*= 9.0 Hz, 2H), 7.26 – 7.12 (m, 3H), 6.93 (d, *J*= 2.3 Hz, 1H), 6.87 – 6.79 (m, 5H). ^{13}C NMR (150 MHz, CD_3OD) δ 158.9, 158.6, 157.4, 156.5, 156.3, 132.1, 130.9, 130.5, 129.0 (×2), 127.0 (×2), 124.0, 123.8, 122.2, 116.6 (×2), 116.5 (×2), 108.2, 98.8, 97.5.

References

1. Jaseer, E.A.; Prasad, D.J.C.; Sekar, G. Domino synthesis of 2-arylbenzo[b]furans by copper(II)-catalyzed coupling of o-iodophenols and aryl acetylenes. *Tetrahedron* **2010**, *66*, 2077-2082.
2. Yao, T.; Yue, D.; Larock, R.C. Synthesis of 2,3-disubstituted benzofurans by the palladium-catalyzed coupling of 2-iodoanisoles and terminal alkynes. *Org. Synth.* **2014**, *91*, 283-292.
3. Lindgren, A.E.; Oeberg, C.T.; Hillgren, J.M.; Elofsson, M. Total Synthesis of the Resveratrol Oligomers (±)-Ampelopsin B and (±)-ε-Viniferin. *Eur. J. Org. Chem.* **2016**, *2016*, 426-429.
4. Iino, T.; Tsukahara, D.; Kamata, K.; Sasaki, K.; Ohyama, S.; Hosaka, H.; Hasegawa, T.; Chiba, M.; Nagata, Y.; Eiki, J.; Nishimura, T. Discovery of potent and orally active 3-alkoxy-5-phenoxy N-thiazolyl benzamides as novel allosteric glucokinase activators. *Bioorg. Med. Chem.* **2009**, *17*, 2733–2743.
5. Liu, J.; Do, T.J.; Simmons, C.J.; Lynch, J.C.; Gu, W.; Ma, Z.; Xuc, W.; Tang, W. Total synthesis of diptoindonesin G and its analogues as selective modulators of estrogen receptors. *Org. Biomol. Chem*. **2016**,*14*, 8927
